# “If we’re here, it’s only because we have no money…” discrimination and violence in Mexican maternity wards

**DOI:** 10.1186/s12884-018-1897-8

**Published:** 2018-06-18

**Authors:** Rosario Valdez Santiago, Luz Arenas Monreal, Anabel Rojas Carmona, Mario Sánchez Domínguez

**Affiliations:** Center for Health Systems Research, The National Institute of Public Health in Mexico, Av. Universidad 655, Col. Santa María Ahuacatitlán, 62100 Cuernavaca, Morelos Mexico

**Keywords:** Obstetric violence, Discrimination, Mexico

## Abstract

**Background:**

Structural and gender violence in Mexico take on various forms, obstetric violence among them. The objective of our study consisted in analyzing experiences of structural and gender discrimination against women during childbirth care at two public hospitals in Mexico.

**Methods:**

We conducted a cross-sectional mixed methods study including a survey of closed questions administered to all women **who received health care** for vaginal or cesarean childbirth at two public hospitals from May 7 to June 7, 2012 (*N* = 512). Those who reported some form of abuse on the part of health-care professionals were then invited to complete a semi-structured interview (20 women agreed to participate). In addition, three focus groups were organized with health-care professionals from both institutions (31 participants): two were composed of nurses and one of obstetrician-gynecologists (OB-GYNs). This work deals with the qualitative component of the study.

**Results:**

The narratives of the health-care professionals interviewed contained expressions of health discrimination relating to certain characteristics of their clients, namely poverty, ignorance, failure to understand instructions and being women. The women, on the other hand, perceived themselves as belonging to a low social class and, as a result, behaved passively with staff throughout their hospital stay. They reported both physical and psychological abuse during care. The first included having their legs manipulated roughly, being strapped to the bed, and being subjected to multiple and careless pelvic examinations. Psychological abuse included reprimands, insults, disrespectful remarks, neglect and scowling gestures when requesting assistance.

**Conclusions:**

The results of our study bear implications for the doctor-client relationship and for the health system in general. They suggest a need to dismantle medical practice – particularly with regard to obstetrics and gynecology - as it has been historically learned and internalized in Mexico. It is imperative to design public policies and strategies based on targeted interventions for dismantling the multiple forms of structural and gender violence replicated daily by actors in the health system.

## Background

The concept of structural violence includes various forms of social vulnerability [1] to discriminatory actions based on differences in social class, physical appearance, ethnicity and gender, among other characteristics. These actions, which normally pass unnoticed, are mirrored in the contrasting treatment of people exhibiting these differences [2].

According to Krieger [3], discrimination is a socially structured and sanctioned phenomenon identifiable by the preeminence of a dominant social group. Justified by the ideology of the dominant actors, discrimination translates into individual and institutional interactions that ensure the privileges of the dominant group. The forms and types of discrimination vary depending on who exerts or endures it. Krieger affirms that comparing the health outcomes of subordinate versus dominant social actors provides only an indirect perspective on the consequences of discrimination.

The study of violence in specific vulnerable groups (e.g., women, minors, refugees and older adults) has traditionally revolved around individual determinants rather than using an explicatory model where the association between structural and other forms of violence can be clearly traced. With regard to women, some forms of violence (e.g., intimate partner violence) have been spotlighted by studies and public policies while others (e.g., femicide) have occurred away from the public eye [4]. A salient example of the latter, obstetric violence [5] has been described as abuse and disrespect during the provision of health-care services [4]. This form of violence is perpetrated by health-care professionals (predominantly by the medical and nursing staff) against pregnant, laboring and postpartum women.

A consensus has not been reached on how to define or measure obstetric violence, with the result that prevalence rates in the literature vary considerably; for Latin America, for instance, reported rates range between 5.4 and 29.1% [6–9].

In recent years, abuse and violence against women during the provision of childbirth services have been classified under typologies including neglect, discrimination, and physical, sexual and verbal violence [5, 10, 11].

However, obstetric violence needs to be viewed as part of a wider picture, not only as a care quality issue. In addition to human rights violations, women victims of obstetric violence [12] experience numerous consequences from these acts. Physically, they undergo practices that range from painful procedures without prior informed consent or anesthesia to injuries and complications resulting from negligence or from excessive medicalization [11, 13]. Psychological consequences have also been reported involving a sense of loss of autonomy, denial of care, and discrimination [12]. These can lead to reduced acceptability and accessibility of obstetric services [14].

Thus far, the study of obstetric violence has centered on the doctor- woman relationship, without considering that this interaction does not occur in a social vacuum, but is intimately linked to expressions of structural and institutional violence which have been legitimized and normalized in the health sector, in public policy and in interpersonal relations overall: expressions which are rooted in the organization of the health system itself and in the education of health-care professionals [15].

Evidence of this can be seen in the configuration of what Good [16] has defined as the medical culture, where different disciplines intertwine (medicine-nursing). Abusive relationships not only exist within the various medical strata, but are learned and internalized from the very onset of a health professional’s education [8, 10, 17].

Castro [18] suggests that a structural link exists between the overall education received by medical students and the authoritarian traits they eventually exhibit in their professional practice. These traits find their most favorable vehicle in the medical habitus [9].

In light of the foregoing, it is clear that the approach to the problem of obstetric violence needs to extend beyond the dominant-subordinate relationship (health-care professionals versus clients). It is important to bear in mind that the power mechanisms underlying the doctor-client relationship have been internalized and legitimized by institutionalized medical practice. They are no longer questioned by the actors (health-care personnel, authorities, or the women themselves in their role as patients). The qualitative approach used in our study enriched our analysis with the voices of health-service users and providers describing the ways in which structural factors shape the manifestations of obstetric violence.

The objective of our study involved analyzing experiences of structural and gender discrimination against women during childbirth care at two public hospitals in Mexico.

## Methods

Our study adopted a cross-sectional mixed methods design featuring a survey of closed questions directed at all the women who received health care for either vaginal or cesarean childbirth at two public hospitals in Mexico, from May 7 to June 7, 2012. Methodological triangulation allowed for embodying prevalence data which, although important, proves insufficient to determine the magnitude of obstetric violence.

As a first step, we administered a questionnaire composed of 34 items on (a) socio-demographic characteristics (10 items); (b) prior obstetric experience (9 items); (c) exposure to violence during the obstetric-care process (11 items); (d) information and consent to medical procedures (31 items); and (e) exposure to intimate partner violence during pregnancy (8 items). The results of the quantitative component have been published elsewhere (Valdez et al., abstract, [8]).

Women who reported some form of abuse on the part of health-care professionals were invited to complete a semi-structured interview. Three focus groups were organized with health-care professionals (two with nursing staff; the other with medical staff, specifically with obstetrician-gynecologists-OB-GYNs) from the same institutions where the women had received health care in a medical setting. This work presents only the qualitative data derived from the interviews with the women and from the focus groups with health-care professionals. Our research protocol was approved by the Research and Ethics Committees of the National Institute of Public Health in Mexico (*INSP* by its Spanish initials). The women and health-care professionals interviewed signed a letter of informed consent prior to participating in the study.

### Population

Health-care users (20 women) and personnel (physicians and nurses from the obstetrics and gynecology department) at two public hospitals in Mexico (31 health-care professionals).

### Instruments

(A) A semi-structured interview guide for exploring how women were cared for in a medical setting during their delivery and postpartum processes; (B) a guide for interviewing nursing personnel; and (C) a guide for interviewing OB-GYNs.

### Procedure

We conducted and audio-recorded semi-structured interviews with the women, whose authorization was obtained prior to the recordings. The interviews were held within 6 to 24 h of delivery in the participating hospital facilities. We also organized three focus groups with physicians and nurses from the obstetrics and gynecology department at the hospitals where the women had been attended to.

Two focus groups were organized with nursing staff, each composed of six individuals. Mostly women, participants were aged 25–52 years, held a bachelor degree in nursing, and had 2–24 years’ work experience.

The focus group with medical staff gathered OB-GYN specialists responsible for attending births in the two hospitals. They included seven women and two men aged 30–38 years and with 1–10 years’ work experience. All the groups, coordinated by two members of the research team, were held in the participating hospital facilities.

### Data analysis

Information from the interviews and focus groups was transcribed and analyzed using Atlas ti V 7 software. Analysis was broken down into five phases: (1) reading of interview and focus group transcriptions to identify codes; (2) discussion and agreements among research team members to define codes; (3) re-reading of transcriptions to establish coding; (4) analysis by code to find regularities and differentials; and (5) elaboration of data concentration tables and matrices based on findings.

Analysis included the processes of open coding, axial coding and construction of a conditional matrix representing the characteristics, consequences and actions pertaining to the phenomenon under study. Having reached the point of theoretical saturation, the team found a conceptual scheme for identifying the central category, or phenomenon around which the other categories were built. The central category was abuse perpetrated by health-care professionals against women, and the related sub-categories were non-consensual care; non-confidential care; undignified medical care; abandonment of care; and health discrimination.

For the purposes of this study, the central category was defined as any action or omission that results in abuse (physical or emotional) or disrespect against women on the part of health-care personnel during childbirth care.

This manuscript discusses the central and the health discrimination categories, both of which clearly reflect the discrimination to which women were submitted by health services during childbirth care.

## Results

### Participants

#### Sociodemographic correlates of abuse

The sample was composed of 512 women: the majority were young adults (aged 13–44 years) dedicated to housework and affiliated with the *Seguro Popular*. They had middle-school education (nine years of schooling), had a partner, and identified themselves as Catholics. Table 1 presents their socio-demographic characteristics by reported abuse.

**Table 1:** Socio-demographic characteristics of abused women (by abuse)

Characteristics	Abuse		Total	*p* value*
	Yes	No		
	(*n* = 138)	(*n* = 374)	(*n* = 512)	
Age (years)				0.610
13–19	26	74	30	
20–24	27	73	34	
25–29	23	77	18	
30–34	38	62	9	
35–39	26	74	7	
40–44	30	70	2	
Education				0.050
None/elementary school	21	79	24	
Middle school	25	75	43	
High school	35	65	28	
University	32	68	5	
Religion				0.503
Catholic	26	74	77	
Other	30	70	18	
None	35	65	5	
Indigenous language				0. 081
Yes	47	53	3	
No	26	74	97	
Marital status				0.321
Currently with partner	26	74	87	
Currently without partner	32	68	13	
Labor status				0.177
Works	30	70	7	
Studies	55	45	2	
Housewife	26	74	85	
Disability	0	100	1	
Does not work	36	64	5	
Insurance				0.477
*Seguro Popular*	27	73	96	
Other	0	100	1	
None	21	79	3	
Number of pregnancies				0.321
1	29	71	45	
2	27	73	26	
3	26	74	17	
4	28	72	7	
5 or more	8	92	5	
Type of childbirth				0.060
Vaginal	32	68	43	
Cesarean	24	76	50	
Scheduled cesarean	18	82	7	

Source: authors elaboration *p value chi square test

### Abuse during childbirth care

Prevalence of abuse reached 29% (*n* = 149), with no differences observed between hospitals (*p* = 0.815). We were only able to interview 20 of the abused women (16 from hospital 1 and 4 from Hospital 2) (Table 2).

**Table 2:** Socio-demographic characteristics of abused women

Característics	Proportion (*n* = 20)
Age (years)	
13–19	39
20–24	39
25–29	11
30–34	6
35–39	5
Education	
None/elementary school	17
Middle school	55
High school	28
Religion	
Catholic	72
Other	28
Indigenous language	
Yes	6
No	94
Marital status	
Currently with partner	72
Currently without partner	28
Labor status	
Student	11
Housewife	89
Insurance	
*Seguro Popular*	100
Number of pregnancies	
1	50
2	33
3	11
4	6
Type of childbirth	
Vaginal	22
Cesarean	78

Source: authors elaboration

The health-care personnel who exerted abuse consisted of nurses (40%), female doctors (30%) and male doctors (30%).

### Health discrimination

Expressions denoting stigmatization and discrimination against women on the part of health-care professionals were identified in the narratives of both women and professionals with regard to the following characteristics: (a) physical appearance, (b) poverty and (c) status as women. Furthermore, the self-perception of belonging to a disadvantaged social class was identified in the narratives of the women as a decisive factor in their submissive behavior towards health-care professionals during delivery care.

### Because they are poor: The voices of health-care professionals

Health-care professionals stigmatized the behavior of poor women (e.g., eating with the food tray on the bed rather than on the table and piling up dirty diapers from their babies on the table) and reported that these actions “drove them crazy.” They also mentioned repeatedly that, because these women were poor, they should make health decisions related to (a) exclusive breastfeeding and (b) contraceptive use. While both proposals are in themselves highly recommended for preserving the health of the mother and newborn, citing poverty as the primary reason for adopting them reflects a discriminatory attitude. Coupled with these comments, the health-care professionals continuously disparaged women for their limited understanding of medical instructions, recommendations and decisions. The following dialogue between a nurse and a woman is an example of this:

According to the nurse, “The mothers here, the majority, are very, what’s the word? Strange. They’re very reluctant to accept the information we give them.”

“On breastfeeding or in general?” inquired the interviewer.

“In general.” replied the nurse. “Here, we place a lot of emphasis on breastfeeding because they always ask for formula milk, but when we give them information, they act very reluctantly.” She was referring to exclusive breastfeeding (Nurses FG).

Health-care professionals argued that the women had difficulty understanding their instructions and requests and that, as a result, their communication was ineffective. They held that the women understood the information at the beginning but quickly forgot it. This was an underlying belief among staff, who described this behavior as annoying and exasperating. They used the phrase “demanding and rude” in their descriptions of these women and attributed their behavior to their “low cultural level” (an expression they commonly used in reference to the women’s low academic level).

They also employed the term “crazy” in reference to their limited schooling (desertion), early gestation (some were adolescent mothers), numerous births, and lack of knowledge about pregnancy and childbirth.

“I think that, here, what this implies, the heart of the matter, is the education of the patients.” (OB-GYNs FG).

“You can explain things to them a lot, but they end up coming back to the same…” (Nurses FG).

“Maybe they understand, but you know, no matter how many times you show them slides, [use] the whiteboard and present [the information], some people are simply blocked. That’s happened to me and it’s exasperating.” (OB-GYNs FG.)

In addition, the health-care professionals repeatedly expressed their annoyance with the demanding attitudes of the women by virtue of their being affiliated with the *Seguro Popular.*^1^ What underlay this complaint, however, was not the affiliation in itself: these women believed that, being poor, they had no right to receive “free” medical care nor to demand that services be of high quality and offer a reasonable level of comfort - as if being poor implied having no rights at all. Once again, stigmatization and discrimination derived from structural social elements such as socioeconomic class were identified and translated into inequalities in care. Furthermore, aspects of the interpersonal relationships between health-care professionals and users marked by entrenched institutional hierarchies and high levels of vulnerability on the part of the users resulted in abuses of power by the former. The following testimony illustrates the points above:

“An angry woman comes up to me and says, ‘Well, you know what? Thanks to me you have work.’ And, although I usually don’t respond, I tell her, ‘Well, thanks to me you have *Seguro Popular,* because I pay my taxes,^2^’ and I didn’t give her the bedpan.^3^ I told her she had already gotten up to bathe, I mean, why did she want a comfortable bedpan when she had already, I mean, when a patient stays in bed it makes things more complicated, so I grabbed it and didn’t give it to her.” (Nurses FG; underlining is the authors’).

In their narratives, the health-care professionals referred explicitly to what was permitted in public hospitals (e.g., multiple pelvic examinations), being teaching hospitals*,* as opposed to private hospitals, where it is understood that women who pay for services will not be examined as many times as “teaching” requires. The following testimony illustrates this contrasting perspective between what in fact *can* be done with women receiving health care at public (poor women) versus private hospitals (women with the means to pay for services). It demonstrates the unequal care provided to women with the same health-care needs but different means and conditions with which to confront them:

“Yes, there are resident doctors (in private medical facilities), yes, but fewer. Why? Because as a private patient, you [the OB-GYN] can’t allow the 15 residents who are still in school to be tampering with her. So the physician in charge of the private patient assigns one [to the case]: ‘Look, man [referring to the resident in training], you’re going to examine her only when necessary. Otherwise, talk to me and I’ll decide what to do, because I get paid 30 thousand pesos’.^4^” (OB-GYNs FG).

### Social-class discrimination: The voices of women

#### Self-perception of social class

Testimonies reflected the self-perception of the women regarding poverty and explained their behavior: in identifying themselves as poor, they did not believe they could protect themselves from the insults of attending personnel. The majority of women interviewed did not see themselves as citizens with rights. Because they had no money to pay for other services, most were affiliated with the *Seguro Popular.* They felt, therefore, that they had no choice but to tolerate the treatment they were given; this, in addition to experiencing a constant fear that something would happen to their babies. Whether from shame or fear of being treated even worse, they did not respond to reprimands from the staff for not being knowledgeable about the physiological processes of infant care, or to their comments concerning their economic situation. Furthermore, the women reported that, having received an education, the professional staff believed they were superior to them and felt they had the right to accentuate that the reason they were giving birth in a public hospital was for lack of money. The following testimony illustrates how one of the interviewees assumed that she had to remain silent and put up with abuse:

“‘So be quiet, because you’re not the only one; there are many of you here.’ [commentary of one physician to a woman]. So I had to be quiet. I couldn’t do anything. We had no money to pay for private services. We had to be there no matter what, right?” (18-year-old first-time mother).

### Abuse of women by health-care professionals

The testimonies of the women revealed basically two forms of abuse: physical and psychological. The health-care professionals reported the same information but did not accept the fact that they were perpetrating violence: they justified their rudeness as necessary for making the women understand instructions and, at times, for saving their lives.

### Physical abuse

The physical abuse reported by the women was characterized by the following actions: having their legs manipulated roughly, being slapped, pinched and strapped to the bed. The following testimony justifies the violent actions exerted against women by health-care professionals:

“Well I tied her to the gurney, grabbed some bandages and put them on her hands and legs. I put them there like that, and that was the only way I could remove the placenta and stop the bleeding. So, to what extent can this be considered an aggression if I’m saving her life? Because I had no other choice but to tie her up so that I could get the baby out, because she wasn’t listening, she wasn’t paying attention to me even though I explained it to her and everything. What do you do [in these cases]?” (OB-GYNs FG).

Physical abuse also translated into poorly practiced routine clinical procedures, for example, sticking women numerous times in the attempt to administer anesthesia or intravenous (IV) serum, performing medical procedures, such as an episiotomy, without anesthesia, and repeating pelvic examinations carelessly and without providing an explanation. Painful in themselves, these are aggravated when conducted without the proper technique. The following testimony details how these procedures were carried out:

The interviewer asked one of the women in the study sample, “Did you know what they were injecting you with?”.

“Well, no,” replied the woman, “it was like...when I asked [they said], ‘it was to speed it up [the delivery],’ but I felt something burning on my back. They had to bend me over and they kept touching my back. The nurse kept on doing it wrong. It would have been better if the doctor did it, right? And again she did it wrong and kept on asking. I mean, they gave me the injection [epidural] three times.” (15-year-old first-time mother).

### Psychological abuse

This form of abuse was characterized by screaming, verbal humiliation, offensive jokes, reprimands for expressing pain or requesting service, scowling gestures and disapproving faces.

Screaming and humiliation were described as a routine form of communication: “You’re not at home;” “You’re not alone, so be quiet!” Ill-treatment extended to the newborns as well: “Do you do this by kilos?” [referring to a large baby], and “This product is for men” [referring to a female baby]. These are vivid examples of the objectification of the female body - even that of female babies.

According to the women’s self-reports, gestures and expressions of disapproval were expressed by OB-GYNs and nurses at various moments during care, particularly when the women requested assistance in climbing onto the gurney, going to the lavatory, and bathing the day after giving birth.

Scowls of disapproval were as severe as stigmatizing of bad mothers, particularly for the unfamiliarity of the women with the physiological processes of pregnancy and childbirth, a lack of knowledge which was exacerbated in the case of adolescent or first-time mothers: “Are you stupid? You don’t know what pregnancy is?”

In addition to the above, it was common practice for health-care professionals to hold the women accountable for any possible adverse results in the health of their newborns. In the delivery room,^5^ allusions to eroticism in the women’s lives were generalized, referring to the sexual enjoyment exclusively of the women, and presuming the moral authority to punish. And there were always those to remind them: “Don’t cry. Deal with it. Remember how you did it. You liked it then and now you’re screaming. So deal with it.” (21-year-old multiparous woman).

The women interviewed reported being repeatedly neglected by staff during their care. Neglect took different forms: from not permitting visits from their families or children, ignoring requests from their relatives, and failing to provide assistance with activities which would have allowed them to move, to not/authorizing the entrance of family members to the women’s rooms at their sole discretion.

The interviewer inquired, “And why are some women allowed to bring a family member with them and you aren’t?”.

“Because she came up yesterday night, the one over there, on that side,” responded the woman, “and she brought a family member to stay with her. But me, no, they brought me up yesterday morning, and yesterday morning they wouldn’t let my mother stay” (21-year-old first-time mother).

In addition to the actions outlined above, neglect acquired particular importance after delivery, when mothers awaited information about their newborns. A number of reports indicated that health-care professionals did not show the babies to the mothers at that time, nor offered information to their family members. Below is one of the most relevant testimonies:

The interviewer inquired, “Did they show you your baby when it was born?”.

“They didn’t show him to me,” responded the woman. “They were checking him. I turned around and looked. They kept on checking him and took him away.” (17-year-old first-time mother).

## Discussion

We identified abusive practices – physical, verbal and discriminatory actions - against women during delivery care at the sampled hospitals. Relating mostly to the social vulnerability of the women, these practices reflect open discrimination against the majority of Mexican women. Our results are consistent with the national, regional and international literature on the topic [7, 11, 19, 20].

Our findings indicate that being poor and holding Seguro Popular (SP) insurance are grounds for discrimination. This bears far-reaching implications, given that the *SP* is the most important public policy ever implemented by the Mexican government to protect the health of the most vulnerable populations. Moreover, the *SP* was designed as a means for reversing segregation in the Mexican health system [21, 22]. According to Link, this form of discrimination in health institutions falls within the category of structural discrimination, as it involves professional health practices undertaken against women within institutional spaces [23].

Our findings provide evidence of violations of individuals’ sexual and reproductive rights, specifically of the right to non-discrimination on social status [24]. The abusive practices observed are opposed to international and national regulations. Internationally, the UN Committee on Economic, Social and Cultural Rights (CESCR) defines health as a “fundamental human right indispensable for the exercise of other human rights” [25].

At the national level, in conformity with Article 4 of the Mexican Constitution, the regulation in force provides that “…every person has the right to health protection” [26]. Furthermore, the Supreme Court of Justice of the Nation established the right to health as a subjective public right, and determined that “the right to health protection pursues, inter alia, the enjoyment of health and social wellbeing services that meet the needs of the population” [27].

Another aspect that warrants particular attention concerns the annoyance expressed by health professionals at the women’s inability to understand the instructions and processes relative to pregnancy and childbirth. They attribute this deficit to ignorance on the part of poor women, without bearing in mind that maternal health literacy involves “cognitive and social skills that determine the motivation and ability of women to gain access to, understand, and use information in a way that promotes and maintains their health and that of their children“ [28]. They overlook the fact that maternal health literacy should be the outcome of quality prenatal care; in other words, that it is not a matter of personal ignorance, but rather a vacuum fostered by inadequate primary-care services.

This chasm between health-care professionals and the population should be deemed is sufficient grounds for reassessing the medical and nursing academic curricula. Established programs should encompass the ethical aspects of health care [10], the interculturality of service [29], and the notion of citizenship in health-care users [30]. The curricula should also provide sensitivity training in the human, sexual and reproductive rights of women [31].

Our findings indicate that health-care professionals are not aware that they engage in practices of violence and discrimination against women. They interpret their behavior as actions that “save lives” and are therefore justified as necessary [11]. Our results in Mexico resemble those obtained in other countries [29] regarding women with specific characteristics including single-mother maternity and ethnicity [32, 33].

Over two million births are attended annually in Mexico [34], the majority in public health institutions, which are the only option accessible to the poorest women. As documented in the present study, approaching these institutions involves having contact with a health system that violates their sexual and reproductive rights, among others.

Subsequent to studies conducted in Mexico [8, 10, 18, 20], decision-makers from the Ministry of Health have implemented a number of strategies to reverse this problem (e.g., the Strategy for the promotion of proper treatment during pregnancy, childbirth and the postpartum period) [35]. However, no evidence is available thus far for identifying the scope and impact of these interventions. Given the complexity of the problem, efforts in this area should be continuous and long-term.

### Limitations

The principal limitation to our study is the fact that our quantitative sample was selected under convenience sampling.

## Conclusions

Our findings support the argument that obstetric violence research must follow an integral approach. Account should be taken of the macro social context, rather than confining analysis to individuals [health-care professionals vs. women demanding obstetric care] who converge in a specific space at a specific time (public hospital-childbirth care).

The encounter between health-care professionals and their clients is conditioned by the characteristics of the health-care system itself. Additionally, it occurs in a social context where not only human relations but also health organizations are permeated by violence [15]. In particular, the naturalization of gender violence against women sustains its reproduction in different contexts; health-care spaces are not an exception. Notwithstanding progress made in information services and the national legal framework, Mexico continues to yield devastating indicators on violence against women including violations of their rights.

In Mexico, as in other countries, health institutions engage in structural discrimination against women. It is expressed in the delivery rooms and by health-care professionals.

## Abbreviations

CESCR: Committee on Economic, Social and Cultural Rights (United Nations)
INSP: National Institute of Public Health in Mexico (by its Spanish initials)
OB-GYNs: obstetrician-gynecologists
SP: Seguro Popular (by its Spanish initials)

## Footnotes

1 A government insurance scheme featuring a service catalog for affiliates (*CAUSES* by its Spanish initials). *CAUSES* is based on agreements with a variety of public and private health-care providers in all the states of Mexico (http://www.seguropopular.org/) last consulted on February 3, 2016).

2 The underlying message in this comment was that, owing to their poverty, poor individuals did not pay taxes. This is technically erroneous, as all consumers pay taxes when they acquire consumer goods.

3 A shallow receptacle for the urine and feces of persons confined to bed, particularly in a hospital.

4 In 2012 equivalent to $ 2308 USD [36].

5 An area in the labor and delivery unit of a hospital where women are assisted during the second stage of labor.
